# Chronic Beryllium Disease: Revealing the Role of Beryllium Ion and Small Peptides Binding to HLA-DP2

**DOI:** 10.1371/journal.pone.0111604

**Published:** 2014-11-04

**Authors:** Marharyta Petukh, Bohua Wu, Shannon Stefl, Nick Smith, David Hyde-Volpe, Li Wang, Emil Alexov

**Affiliations:** 1 Computational Biophysics and Bioinformatics, Physics Department, Clemson University, Clemson, South Carolina, United States of America; 2 School of Nursing, Clemson University, Clemson, South Carolina, United States of America; 3 Department of Chemistry, Clemson University, Clemson, South Carolina, United States of America; University Medical Center Freiburg, Germany

## Abstract

Chronic Beryllium (Be) Disease (CBD) is a granulomatous disorder that predominantly affects the lung. The CBD is caused by Be exposure of individuals carrying the HLA-DP2 protein of the major histocompatibility complex class II (MHCII). While the involvement of Be in the development of CBD is obvious and the binding site and the sequence of Be and peptide binding were recently experimentally revealed [1], the interplay between induced conformational changes and the changes of the peptide binding affinity in presence of Be were not investigated. Here we carry out *in silico* modeling and predict the Be binding to be within the acidic pocket (Glu26, Glu68 and Glu69) present on the HLA-DP2 protein in accordance with the experimental work [1]. In addition, the modeling indicates that the Be ion binds to the HLA-DP2 before the corresponding peptide is able to bind to it. Further analysis of the MD generated trajectories reveals that in the presence of the Be ion in the binding pocket of HLA-DP2, all the different types of peptides induce very similar conformational changes, but their binding affinities are quite different. Since these conformational changes are distinctly different from the changes caused by peptides normally found in the cell in the absence of Be, it can be speculated that CBD can be caused by any peptide in presence of Be ion. However, the affinities of peptides for Be loaded HLA-DP2 were found to depend of their amino acid composition and the peptides carrying acidic group at positions 4 and 7 are among the strongest binders. Thus, it is proposed that CBD is caused by the exposure of Be of an individual carrying the HLA-DP2*0201 allele and that the binding of Be to HLA-DP2 protein alters the conformational and ionization properties of HLA-DP2 such that the binding of a peptide triggers a wrong signaling cascade.

## Introduction

Chronic Beryllium Disease (CBD) is a pulmonary granulomatous disorder caused by an immune reaction when individuals are exposed to beryllium (Be) [2]. About 18% of people who are exposed to Be in the workplace may develop CBD depending on a number of risk factors such as their genetic susceptibility, the duration, the concentration of the Be exposure, and their smoking habits [3], [4], [5], [6], [7]. Out of these risk factors, the genetic susceptibility of the individuals is shown to be the dominant contributor in the development of CBD [2], [8], [9]. Understanding the molecular mechanism of CBD would help prevention and treatment of disease [10], [11], [12], [13], [14].

The Major Histocompatibility Complex II (MHC II) controls the activation of the immune system in response to foreign microorganisms. The first component in this process is the binding of small peptides (that are part of an antigen) to the MHC II. The second component is the conformational change of MHC II induced by the peptide binding which makes the MHC II complex “recognizable” by T-cell receptors along with the subsequent activation of T-cells (TCs) [9], [15], [16]. Different conformational changes result in the activation of different TCs and thus lead to different immune responses. These conformational changes are caused by the binding of variety of peptides to alleles of MHC II. In addition, the associations of MHC II alleles with various diseases have been previously reported [17], [18], [19].

Of particular interest for this study is one of the MHCII molecules, the HLA-DP2 (coded by DPA1*0103, DPB1*0201), which has been shown to be associated with development of CBD [3], [20], [21]. By analyzing various HLA-DP molecules of the MHCII and focusing on the amino acid sequence forming the peptide binding pocket, it was demonstrated that approximately 85% of CBD patients have a glutamic acid residue at position 69 in the β-chain (βGlu69) of HLA-DP [20], [22]. In other HLA-DP proteins, position 69 is taken by a lysine (Lys) amino acid. Because the side chain of Glu residue is shorter than of Lys, it can be speculated that the binding pocket of HLA-DP2 is wider comparing with the other HLA-DP.

The selectivity of HLA-DP2 towards different peptides is typically analyzed by reviewing the corresponding binding pockets for each amino acid of the peptide. Typically these pockets are labeled according to the residues in the peptide, i.e. pocket p1 refers to the protein pocket where the side chain of the first amino acid of peptide will be accommodated. Some of these pockets are very specific, others are not. Thus, the crystal structure of HLA-DP2 with a self-peptide derived from the HLA-DR α-chain (pDRA) [23] reveals that HLA-DP2 has four pockets which are able to bind variety of amino acids [4], [23], [24], [25]. Thus, the p1 pocket prefers hydrophilic residues, p2 can accommodate positively charged residues, p6 could be occupied with aromatic residues, and p9 might accept larger residues. A molecular docking approach discovered that p1, p2 and p6 peptide positions are the most important for binding to the HLA-DP2 protein [24], [26]. Comparing HLA-DP molecules with the HLA-DP2, the replacement of Lys69 with Glu69 (pointing toward peptide position 7, p7) and the other two glutamic acid residues, βGlu68 and βGlu26 (pointing toward p4 and p6), on HLA-DP2, results in an acidic pocket that might potentially accommodate the Be ion [1], [23], [26]. The existence of such an acidic pocket, providing a strong negative potential, was the reason for the speculation that even an endogenous self-peptide with Be can bind to HLA-DP2 and be recognized by the T cells [26]. Thus a recent *ex vivo* study demonstrated that some endogenous self-peptides are able to bind with HLA-DP2 in the presence of Be [26]. When these peptides were exposed to the HLA-DP2 protein, Be-responsive TC receptors were able to recognize those peptides and stimulate the accumulation of cytokines by TCs. Another study examined 40 human peptides for their ability to stimulate the secretion of interleukin-2 (IL-2), a type of cytokine signaling molecule secreted mainly by CD4+ and CD8+ T-cell, *ex vivo*, and 11 of them were found to initiate production of IL-2 by TCs in the presence of the Be ion [26].

**Figure 1:**
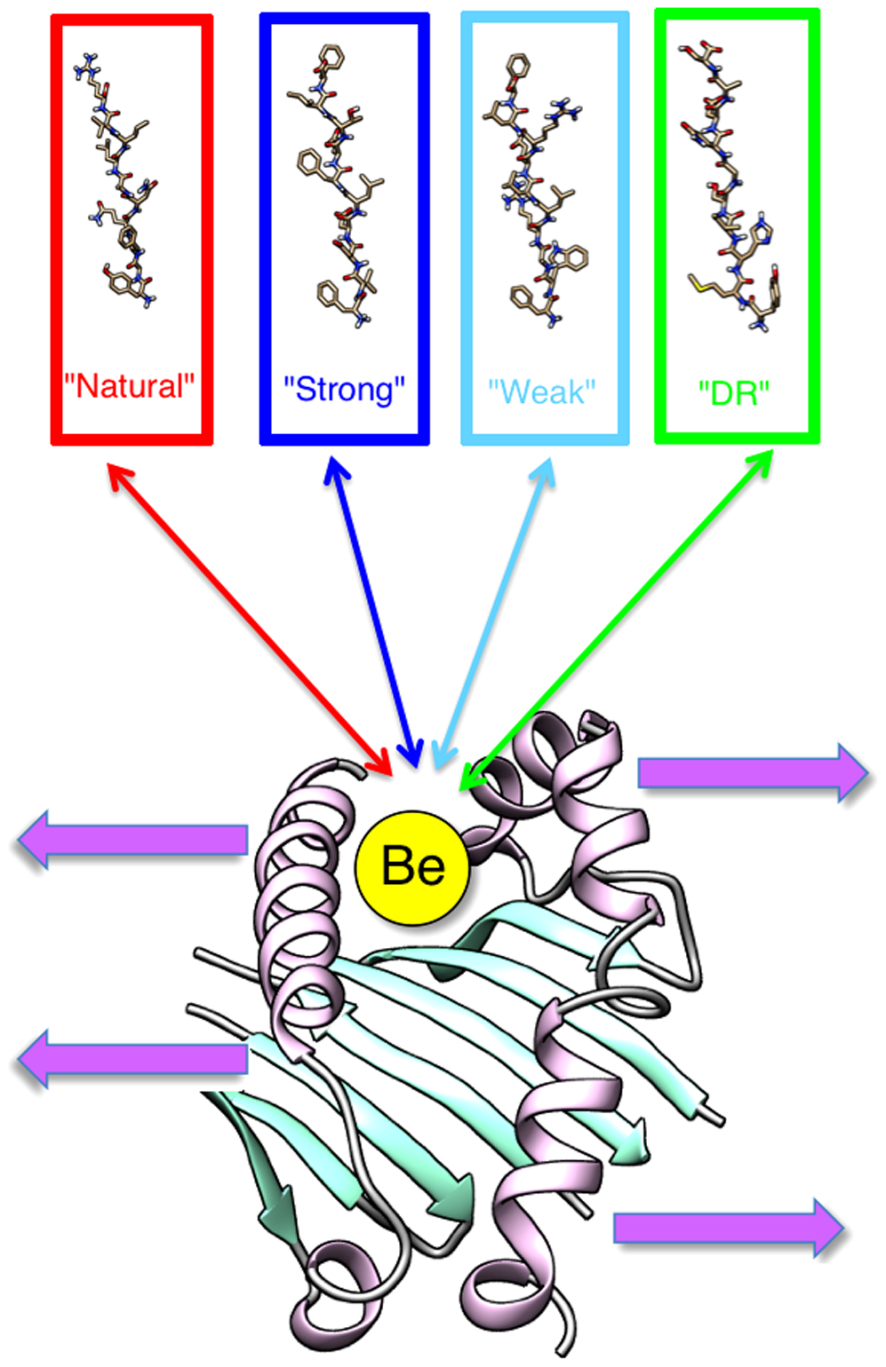
Graphical depiction of our investigation of the effects of the Be ion on the complex made up of a HLA-DP2 protein, the Be ion, and a small peptide. We investigated four types of small peptides (“Natural”, “Strong”, “Weak”, and “DR”) and two binding scenarios: (1) the ion bound to a small peptide and (2) the ion bound to the HLA-DP2 protein. Effect predictions include binding affinity, conformation changes of the peptide binding pocket, pKa shifts of titratable groups of the protein upon peptide and/or the Be ion binding.

Although, in multiple papers the Be ion is suggested to bind directly to HLA-DP2 [27], [28], only recently it was experimentally shown [1] that Be binds first and then the peptide binds to the HLA-DR2-Be complex. However, the reason for this sequence of events is not clear and here we provide plausible explanation based on binding energy calculations. In addition, it is not clear if the sequence of the binding events causes different conformational changes of the HLA-DR2-Be-peptide complex. In this work, we investigate *in silico* both scenarios: (a) peptides which bind to the protein with the Be ion placed inside the protein pocket which induces conformational changes in HLA-DP2 that are necessary for its recognition by TCs; and (b) the Be ion binds to the peptide first and thus changes the peptide-binding specificity and affinity to HLA-DP2 resulting in conformational changes in the HLA-DP2 that are necessary for its recognition by TCs. This is done by analyzing the changes in binding affinity and conformational changes of the protein upon binding the four sets of small peptides (Figure 1): peptides that are known to cause the activation of TC receptors in normal immune response; peptides that prefer to bind to HLA-DR but not HLA-DP; and peptides that are known to bind to the protein in the presence of the Be ion and induce the production of high/low concentration of inflammation cytokines in TCs that are the cause of the autoimmune disease [26]. It is anticipated that by comparing the effects of each set of peptides we will be able to reveal additional details of molecular mechanism of CBD along the finding of recent experimental work [1].

## Methods

**Figure 2:**
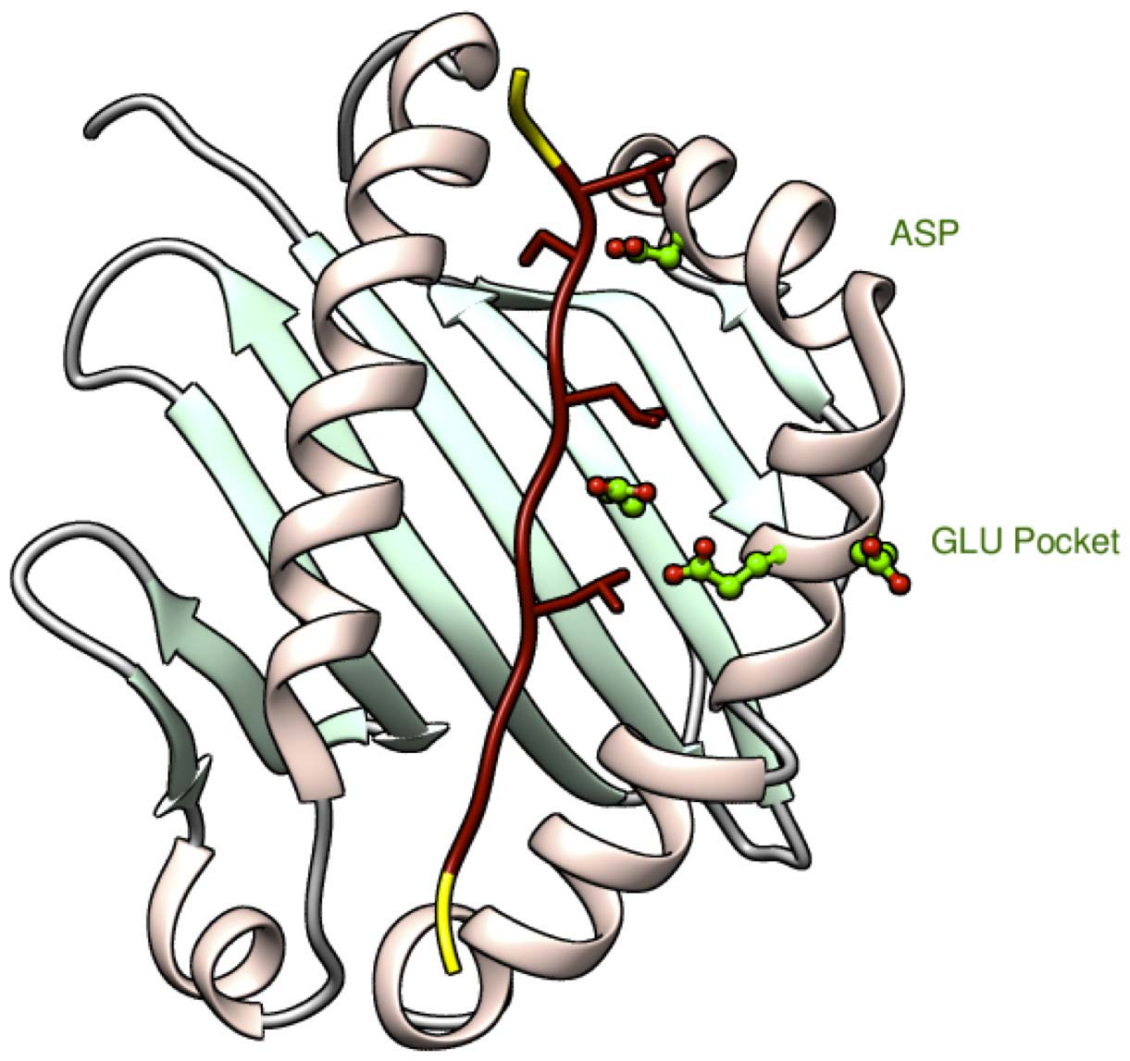
Illustration of the two investigated binding scenarios. In the top panel (A) the Be ion is in complex with the protein and then the peptides were added, peptide→(Be+protein) scenario; and in the bottom panel (B) the Be ion is in complex with the peptides and then the protein is added, (Be+peptide)→protein scenario.

This section describes the selection of peptides to be investigated and the methods for the 3D structure modeling, the Be ion binding site prediction, the molecular dynamics (MD) simulations, the geometry analysis, the binding free energy and pKa calculations. In each of the cases, two scenarios will be investigated: (a) the peptide binding to the (Be-HLA-DP2) complex and (b) the Be bound first to the peptide resulting in a (Be-peptide) complex, then the Be-peptide complex binds to the HLA-DP2 protein (see Figure 2, panels A and B respectively).

### Selection of peptides to be investigated

To investigate the specificity of peptides to HLA-DP2, four different classes of peptides were selected (Figure 1): (1) 10 peptides that bind to the HLA-DP2 were selected from the 40 human peptides based on their ability to stimulate the immune response detected experimentally as an increase of the IL-2 secretion by more than 15 pg/ml in the Be-specific T cell hybridomas AV22 and by 45 pg/ml in the Be-specific T cell hybridomas AV9, which were termed “strong” peptides [26]; (2) 10 additional peptides were selected from the same 40 human peptides which were also found to increase the IL-2 secretion but not as much as the peptides in the “strong” class (the increase of the IL-2 secretion was less than 6 pg/ml in both Be-specific T cell hybridomas AV22 and AV9); these were termed “weak” peptides; (3) 10 peptides which were bound to HLA-DP2 and known to trigger the immune system reaction without the Be ion were chosen and were termed “natural” peptides [5]; and (4) 10 peptides that naturally bind to HLA-DR which were randomly selected from syfpeithi database (http://www.syfpeithi.de) and were termed “DR” peptides. This last class was created because the HLA-DR structure is similar to the HLA-DP2 structure [6], which provides the opportunity to compare the physical properties of peptides binding to HLA-DR and HLA-DP2. All peptides in each class were cut to a length of 10 amino acids. In investigating the molecular mechanism of CBD, it is anticipated that peptides belonging to different classes may have a different affinity and may induce different conformational changes. For example, peptides from the “strong” class may have the highest affinity to bind to HLA-DP2 in the presence of Be and induce conformational and pKa changes distinctly different from peptides in the “natural” and “DR” classes. The binding of peptides in the “weak” class may cause effects similar to the “strong” peptides, but the magnitude of these changes is expected to be smaller. Alternatively, it can be envisioned that the presence of the Be ion in combination with the HLA-DP2 molecule is the leading factor and any peptide will cause CBD.

### Structure modeling

All of the above selected peptides are of various lengths which could introduce artifacts into the computational modeling. So, this is why the lengths need to be made the same. Since the experimentally studied peptides (the “strong” and “weak” classes) are of length 10 [23], [26], the other “natural” and “DR” peptides were also trimmed to a 10 amino acid length. The trimming was guided by preserving the interactions of peptide residues with the key amino acids in the HLA-DP2 protein [9], [23], [26].

The crystal structure of HLA-DP2 was retrieved from the Protein Data Bank (PDB) (http://www.rcsb.org), PDB ID: 3LQZ. It contains an α chain, a β chain, two ligand chemical components, and it shows HLA-DP2 crystalized with a bound natural peptide of 15 amino acids. We chose to include the 10 residues of the crystallized peptide that were located completely within peptide binding pocket of HLA-DP2 (shown in red in Figure 2): peptide residue numbers 1-10 (sequence FHYLPFLPST), to be consistent with Ref. [1]. Our decision of where to cut the peptide was reinforced based on the interaction of the peptide with the key residues in the protein. We predict that the positively charged Be ion will bind to the protein near the negatively charged pocket formed by the three glutamic acid residues in the protein (shown in green in Figure 2) at positions 68, 69, and 26 [20], [23], [26], in accordance with recent experimental work [1]. Using the crystallographic structure, it was found that the peptide residue numbers 4 and 7 are in close proximity to the putative Be binding site and because of this, these two residues are considered to be the key positions in the peptide. When choosing where to cut the other “natural” and “DR” peptides, several considerations were made. First, we wanted to keep the key peptide positions, p4 and p7, and secondly we wanted to keep the peptide residue number 10 as well. The reason for keeping peptide residue number p10 is the following. In the HLA-DP2 protein there is another key residue, an aspartic acid, at position 55 (also shown in green in Figure 2) that is expected to form hydrogen bonds with the peptides or the T-cell receptor. In the crystallized natural peptide, possible interactions between the peptide and this aspartic acid corresponds to a polar tyrosine at peptide amino acid p10, making this position also a key residue in the peptide. Thus, the final selection was made to consider the peptide segment from residues 1-10 as a common 10 amino acid length segment for the modeling of all the peptides.

The 3D structures of the “strong”, “weak”, and other “natural” peptides are not available in neither free form nor bound to HLA-DP2 and therefore have to be modeled (note that the structure of HLA-DP2-Be-peptide complex [1] was not available prior the manuscript was submitted for review). We used the peptide in PDB ID: 3LQZ as a template and have built models for the rest of “natural”, “strong”, and “weak” peptides bound to HLA-DP2. In doing so, several considerations were made (all alignments are available in Material S1). The alignment of the other natural peptides to the 10 amino acid positions of the crystallized peptide template were based mainly on maintaining the biophysical characteristics of key peptide positions 4, 7, and 10. Additionally, we also tried to maintain the biophysical properties of the remaining 7 positions. For example, at positions 1 and 6 in the crystallized peptide, there are hydrophobic phenylalanine residues. Thus, in other peptides, we tried to conserve the biophysical characteristics of these positions by aligning the other peptides to have hydrophobic residues at positions 1 and 6. Similar considerations were made for the alignment of the “strong” and “weak” peptides to the template peptide.

The alignment and cutting of the peptides that bind to HLA-DR (“DR” peptides) was done in a similar fashion. We started with the crystallized structure of HLA-DR with a bound peptide (PDB ID:1KG0). We then used the crystallized DR peptide as a template for aligning and cutting other “DR” peptides. Because we intended to use these peptides as a control (they do not normally bind to HLA-DP2), we first aligned the peptide crystallized with DR to the same peptide template used for the “natural” peptides (taken from PDB ID: 3LQZ) to increase the likelihood of a peptide that naturally bind to HLA-DR to bind to HLA-DP2. We did the alignment in the same way (aligning residue positions 4, 7, 10, and then other positions) and cut the crystallized HLA-DR peptide to include the amino acids 1-10 (sequence YVKQNTLKLA). (The fact that the DR-peptide was also cut to include positions 1-10 was coincidental; the alignment was based entirely on biophysical characteristics). After the “DR” peptide was aligned to and cut based on the HLA-DP2 peptide, we then used it as a template for cutting the other 10 “DR” peptides. The alignment was done in the same fashion as stated before for the HLA-DP2 natural peptides. To create the three-dimensional structures of the peptides, we used the crystallized peptide as a structural template and mutated each residue individually via side chain replacement using VMD [29]. Once all of the peptide structure files were created, we then created additional structure files containing the ion, protein, and peptide to represent the two different binding processes. For the binding processes, we first identified the appropriate binding site of the Be ion on both the HLA-DP2 protein and the peptides.

### Placement of the Be ion on the surface of HLA-DP2 and the peptides

**Figure 3:**
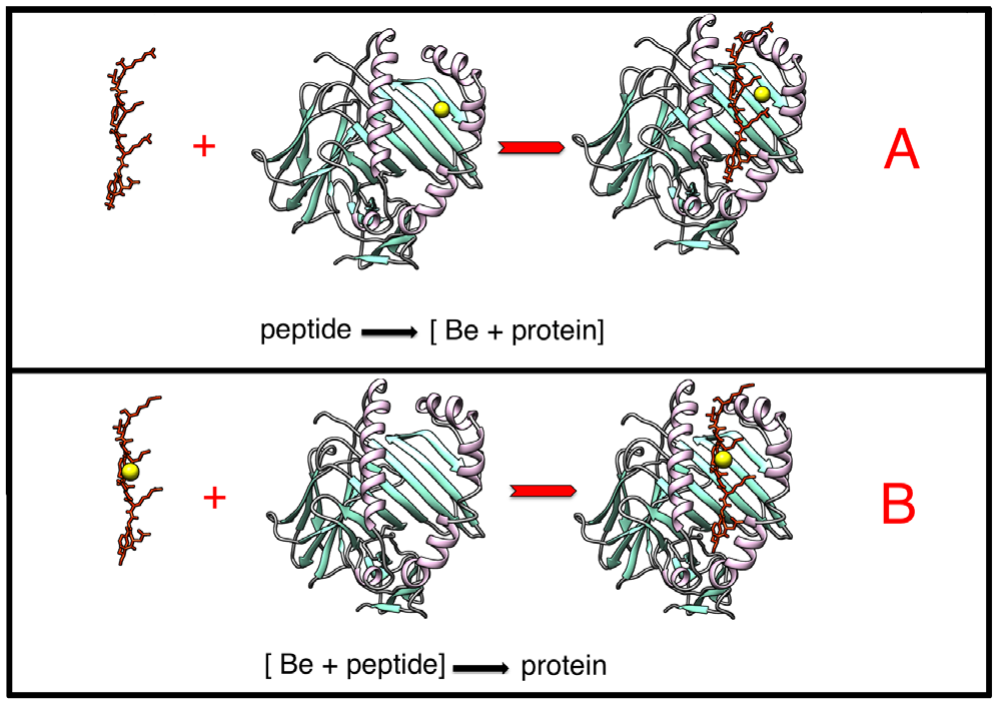
The HLA-DP2 binding pocket in complex with the crystallized natural peptide of 15 residues. The 10 residues of the peptide used as a cutting template are shown in red with important residues having extended structure. The removed residues are in yellow. Key residues in HLA-DP2 are shown in green; the aspartic acid is labeled as *ASP* and the three glutamic acid residues are labeled as *GLU Pocket*.

To investigate the sequence of forming the (Be-HLA-DP2-peptide) complex, we modeled two different scenarios (Fig 3). This is done for computational purposes to compare the energy components and conformational changes, while not addressing questions if this occurs intracellularly or on the cell surface.

- the Be ion is first bound to the HLA-DP2 protein forming a (Be-HLA-DP2) complex, and then the peptides bind to the (Be-HLA-DP2) complex (in text described as “peptide→(Be+protein)” scenario); This requires predicting the Be binding site on HLA-DP2 in the absence of the peptides.
- the Be is first bound to the peptide forming a (Be-peptide) complex, that binds to the HLA-DP2 protein (in text described as “(Be+peptide)→HLA-DP2” scenario). This requires predicting the Be binding site on the free peptides.

It can be expected that the Be ion binds to the HLA-DP2 or to the peptide surface non-specifically, and that the electrostatic force favors this binding. To determine the position of the positively charged Be ion on both the surface of the HLA-DP2 protein and on the exterior of the small peptides that bind to the HLA-DP2 protein, we utilized the BION webserver (http://compbio.clemson.edu/bion_server/) [30]. The algorithm implemented in BION relies on a DelPhi [31] generated potential map in conjunction with an in-house clustering algorithm [32]. The predictions take into account the magnitude of the electrostatic potential at selected surface-bound grid points and the biophysical properties of the ions such as radius and charge. The representative grid points are sorted in descending order (by absolute value) of the potential and the position of a given point within this list is termed Rank. Only the first ranked plausible ion position was used for further analysis. Note that Be ion binding sites for each unbound peptide were independently predicted and are slightly different depending on peptide sequence and conformation.

### Placement of peptides onto the protein

Once the Be was placed onto either the protein or the peptide, we then placed each of the 40 peptides separately into the HLA-DP2 protein using Chimera [33]. Each peptide was placed in such a way as to be as similar as possible to the placement of the natural peptide found in the PDB file. We used the coordinates of the crystallized HLA-DP2 peptide found in the PDB file as the initial position of all the other peptides (“natural”, “strong”, “weak”, and “DR”) when binding them to the HLA-DP2 protein. When there were clashes between the side chains of the peptide and the side chains of the HLA-DP2, we manually adjusted the position of the entire peptide in order to very slightly lift the peptide out of the peptide binding pocket of HLA-DP2 or tilt the peptide within the pocket until the side chains did not overlap. In the case where the peptide side chains overlapped with the ion radius, side chain rotamers defined in the Chimera rotamer library were used to eliminate the overlap. All structures are available for download from http://figshare.com/articles/Chronic_Beryllium_Disease_Revealing_the_role_of_beryllium_ion_and_small_peptides_binding_to_HLA_DP2/1147422.

### Energy minimization and MD simulations

Once the structures were built, the protein, peptides and Be ion were relaxed and simulated using NAMD [34]. Each case was split by chain and protonated using the Charmm22 force field. These protonated chains were then recombined and an explicit water sphere was created around the complex using VMD with the TIP3P water model [29]. This sphere was created with a padding of 15 Å around the protein. To avoid initial structural clashes this structure was first relaxed by NAMD software [34] for 5000 steps via its conjugate gradient algorithm, and then, the minimized structure was simulated at a temperature of 298K for 1 nanosecond using 2 femtosecond time steps. The simulation used spherical boundary conditions to contain the complex and Langevin dynamics to incorporate random forces and damping of motion into the run. Every 100 steps, a snapshot of the protein was exported to a DCD trajectory file that was later unzipped into multiple PDB snapshots.

### RMSD and pocket distance analysis

**Figure 4:**
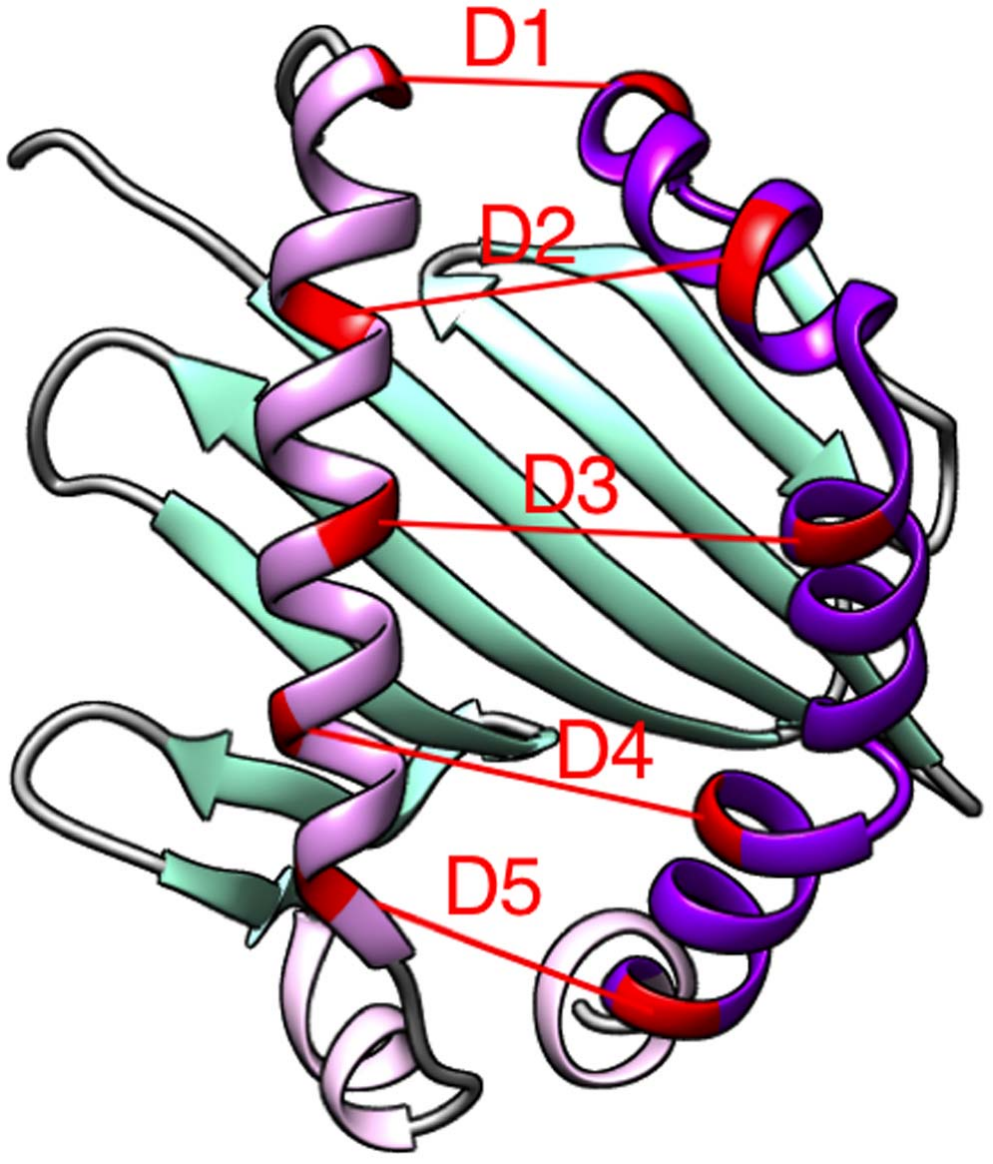
Cartoon representation of HLA-DP2 protein (PDB ID 3LQZ). Peptide binding pocket is shown in light and dark purple. Residues taken into account for distance calculations are in red. Change in RMSD due to peptide/Be binding was analyzed for residues in dark purple. Illustration was made with Chimera software [33].

Once the results were obtained from the MD simulation, the snapshots of each of the cases were utilized for the screening of conformational changes of the Be binding pocket of the protein as a function of time. These changes in HLA-DP2 pocket due to the binding of small peptides and the Be ion were analyzed in two ways:

1. The change in RMSD of residues 50–86 of chain B (Figure 4, labeled in dark purple). Our preliminary data suggests that among two helices (one formed by chain A residues, and another by chain B residues) that form the peptide-binding pocket of HLA-DP2, the helix that is formed by chain B residues experiences the primary conformational transformation upon interacting with the “natural” peptides (not presented).
2. In order to depict the process of the opening-closing of the pocket, we observed the change in five different distances between pairs of residues between the helices forming the peptide-binding pocket of HLA-DP2 (Table 1, Figure 4, labeled in red).

**Table 1:** Selected distances between residues forming peptide-binding pocket of HLA-DP2.

Distance label	Residue 1	Residue 2
1	Arg76.A	Glu50.B
2	Leu70.A	Tyr58.B
3	Ile65.A	Asp64.B
4	Leu60.A	Met76.B
5	Ala56.A	Leu83.B

For statistical significance, we used the peptide class representative RMSD and the distances between five pairs of residues that were calculated as the median RMSD values, and as the mean of distance values respectively within each case in the group.

### Binding energy calculations

The binding free energy of the energetically-minimized protein complex was calculated via the following [35], [36], [37], [38]:
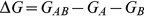
(1)where 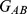 is the free energy of the complex; 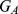 and 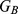 are energies of A and B parts of the complex. Only the electrostatic – Coulombic (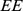) and polar component of solvation energy (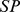) – were calculated using the Delphi software (scale was 1 Å per grid, perfil 70%, dielectric constants for protein and water were 1 and 80 respectively) [31] and van der Waals (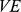) energies, calculated with NAMD [34] (we used the data from one step of the minimization of complex and each of its partners), were taken into account in order to calculate the free energy of the complex and each of its parts:
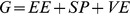
(2).

Such an approach, with slight variations, was previously applied by us to model the changes of the binding free energy [35], [36], [37], [38] and a variation of this method was benchmarked against experimental data [39]. For “(Be+peptide)→protein” scenario (Be ion bound first to the peptide), the binding energy was calculated between the protein (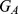) and the complex of the peptide and the Be (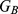). For the “peptide→(Be+protein)” scenario (Be attached to the protein first), we analyzed the binding affinity between the protein (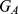) and the Be-peptide complex (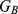). We also calculated the binding free energy between peptides (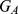) and HLA-DP2 protein (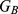) when modeled without the Be ion. In both cases, *G_AB_* refers to the energy of the entire complex Be-HLA-DP2-peptide. It should be pointed out that the current implementation of eq. (2) does not use weighted coefficients and thus overestimates the energy changes, as outlined in another study of protein folding free energy [40]. Due to this, the results obtained using eq. (1) are used for ranking only, not to infer absolute binding free energy changes.

### pKa calculations

We also performed pKa calculations for the ionizable groups within these complex structures and investigated the effects of the presence of the Be ion. The pKa values were obtained with Multi-Conformer-Continuum-Electrostatics (MCCE) program [41], [42], [43]. MCCE calculates the equilibrium state of each conformation and the charged state of the ionizable residues. It simulates the conformational and ionization changes in a Monte Carlo procedure and couples the protonation events with the conformational changes. The calculation was done as a function of pH with an internal dielectric constant of 4.0. The Be ion topology file was created with the parameters: radius = 1.53 Å, charge = +2.0e, and reference energy = −18.430 Kcal/mol.

The pKa calculations were performed on 40 bound complex structures in absence of Be ion (protein+peptide) along with 80 structures with the Be ion (40 structures of the peptides bound to the Be-HLA-DP2 complex and 40 structures of HLA-DP2 to bind with the Be-peptide complex). MCCE predicted the pKa value of the ionizable residue of each structure, and the pKa shifts were analyzed and discussed, with emphasis on the role of the Be ion and the binding.

## Results and Discussion

At normal conditions, MHCII controls the activation of the immune system in response to foreign microorganisms. Its proteins bind a very specific range of exogenous peptides to the peptide-binding pocket and undergo some conformational changes that are recognizable by various TC receptors. However, in patients with CBD, high concentration of Be ions was shown to influence HLA-DP2 activity and resulting in the initiation of “false” signal cascades that cause the development of the chronic autoimmune disease. In order to investigate the molecular mechanism of the HLA-DP2 malfunction in the presence of Be ions, we investigate two scenarios: peptide→(Be+protein) and (Be+peptide)→protein, along with the binding of four different sets of peptides (“natural”, “DR”, “strong”, and “weak”, see Methods section for details). The goal is to analyze the changes in the key processes of the initial immune response associated with HLA-DP2 (peptide binding and subsequent protein conformational changes) for all cases. It is important to emphasize that the binding site of TC receptors is on the same side of the peptide-binding pocket of HLA-DP2, and thus the “false” signal cascade initiation depends mostly on the conformational change of the protein, and the strength of peptides binding to the protein might be less important for the process of the complex recognition by TC receptors. As the manuscript was under review, this hypothesis was experimentally confirmed and it was shown that TC receptor recognizes the conformational changes in the surface of DP2-peptide complex induced by the internally bound Be ion [1].

### Binding affinity of small peptides to HLA-DP2 protein

If the immune response is related to the binding affinity of the corresponding peptides to HLA-DP2, then one can expect that the peptides in the “strong” set should have the strongest affinity in the presence of the Be ion, and this affinity should be similar to the affinity of the “natural” set of peptides in the absence of the Be ion. The Be ion should favor binding of peptides in the “weak” set to the protein as well, however, the binding affinity should be smaller than for peptides in the “strong” set. Furthermore one expects the “DR” peptides to have the lowest or no affinity to HLA-DP2, in both the presence and in the absence of the Be ion. Although the binding affinity may not be a key factor, as indicated above, the binding free energy estimations may address the question of which scenario is more energetically favorable, the scenario “peptide→(Be+protein)” or “(Be+peptide)→protein”.

**Figure 5:**
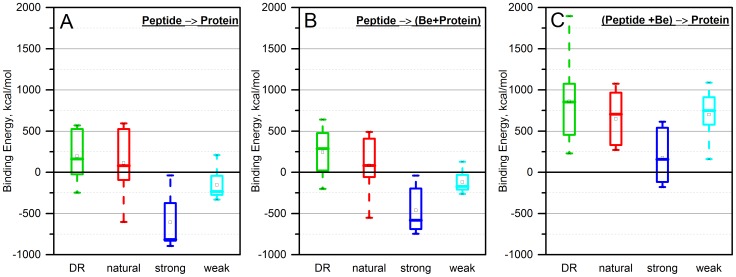
Binding free energy between A) peptides and HLA-DP2 protein without the Be ion; B) peptide and complex of HLA-DP protein with the Be ion in case of peptide→(Be+protein) scenario; C) complex of peptides and the Be ion with the protein for (Be+peptide)→protein scenario.

Figure 5 shows the calculated binding free energy (eq.1) after 5000 steps of minimization. The grouped box chart was created based on the data for all of the peptides within the corresponding set (“strong”, “weak”, “natural”, and “DR”) and based on the model for placing the Be ion in the complex (“peptide→(Be+protein)” and “(Be+peptide)→protein”).

The immediate observation can be made by comparing Figure 5.B and C: The scenario “(Be+peptide)→protein” is less energetically favorable than the “peptide→(Be+protein)” scenario. This is due to several reasons. The peptides themselves, including “strong” and “weak” peptides, do not have an acidic cluster, i.e. do not have a binding pocket with a strong negative potential which can attract positively charged Be ions. In addition, free peptides are expected to be quite flexible and therefore are unable to keep tightly bound ions.

Comparing Figure 5.A (binding without the Be ion) and Figure 5.B (binding with the Be ion), no significant changes are predicted for each of the peptides, however, “strong” peptides are predicted to have the highest affinity. The reason for this prediction will be discussed in the *pKa* section below. Here, we simply mention that the formation of the acidic cluster of three Glu residues affects the protonation states of these Glu residues in both the free HLA-DP2 and in the bound HLA-DP2 protein. These changes are not taken into account in the protocol that calculates the binding free energy. This was done to study the binding, conformational, and ionization changes separately.

Further analysis of Figure 5 reveals that the binding affinities are ranked in accordance with our expectations: the peptides in the “strong” set are the strongest binders, followed by “weak”, “natural”, and “DR” peptides. As we have indicated above, the binding free energies are for ranking only and it is quite possible that “DR” peptides do not bind at all to HLA-DP2.

### Conformational changes in HLA-DP2 upon small peptides and/or Be ion binding

The binding of the peptide and the Be ion to HLA-DP2 should trigger conformational changes that are distinctly different from the conformational changes induced by “natural” peptides in the absence of Be. This is what will initiate the “false” signaling cascade and is the cause of CBD. We assume that these conformational changes should be caused by distinct different conformational changes within the peptide binding pocket as well. Thus, 1 ns MD simulations were conducted and later analyzed to observe the changes in the RMSD of one of the “walls” that form the peptide-binding pocket (helix 50–86.B) and the width of the binding pocket via the monitoring of five selected distances between the CA atoms of the residues that form this pocket (Figure 4). The distance and RMSD fluctuations were analyzed for the last 0.4 ns.

#### 1. RMSD analysis

Because the initial conformation of HLA-DP2 is the same for all cases (before the energy minimization steps), it allowed us to monitor how it will change over a period of time while binding different types of peptides and/or the Be ion. Here, we are focusing on the changes associated with helix 50–86.B (residues 50–86 of chain B), which forms one of the walls of the binding pocket and is expected to play an important role in the peptide and Be binding by HLA-DP2 and is expected to undergo conformational changes which are different from the changes observed for “natural” peptides in the absence of the Be ion.

**Figure 6:**
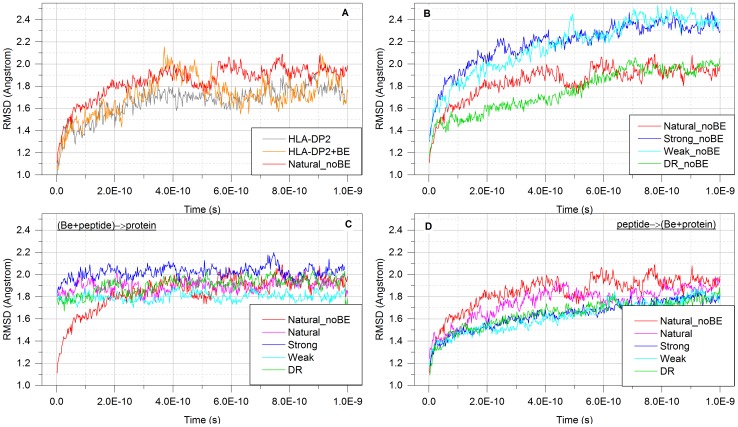
Change in RMSD of one of the walls of the peptide-binding pocket of HLA-DP2 during MD simulations in presence of peptides and/or the Be ion.

In order to have a reference to compare with, the initial simulations were done on the unbound HLA-DP2, on HLA-DP2 with natural peptide, and on HLA-DP2 with the bound Be ion (Figure 6.A). One can see that in the unbound HLA-DP2, the helix 50–86.B undergoes some conformational fluctuations and reaches saturation at about 1.8 Å. The same simulations that were performed with “natural” peptides result in larger fluctuations, reaching RMSD saturation at about 2.0 Å. The simulations done on HLA-DP2 with the bound Be indicate that Be increases the magnitude of the fluctuations (±0.1 Å), but the saturation RMSD is almost identical to the HLA-DP2 without Be. Based on these results, it can be speculated that the binding of “natural” peptides to the HLA-DP2 protein causes the helix 50–86.B to deviate further from its crystallographic position and thus to open the peptide binding side. These changes can be recognized by specific TC receptors and trigger the “normal” cascade of signals.

Figure 6.B shows the RMSD without the Be ion present. It can be seen that “natural” and “DR” peptide binding causes almost the same RMSD on the HLA-DP2 complex, while the binding of “strong” and “weak” peptides in absence of Be causes a much larger RMSD of helix 50–86.B. The direction of the change, the increase in the RMSD upon the binding of the peptides, is the same as for binding “natural” peptides to HLA-DP2, and perhaps will initiate the same immune cascade.

The simulations performed with all the sets of peptides (“DR”, “natural”, “strong”, and “weak”) with the Be ion in the scenario “(Be+peptide)→protein” indicate that there is practically no difference between the RMSD of the complexes (Figure 6.C). This is similar to the RMSD induced by the Be ion alone and is in agreement with the binding free energy calculations (Figure 5). Indeed, all peptides within scenario “(Be+peptide)→protein” were calculated to bind much more weakly than “normal” peptides in the absence of the Be ion. This is the reason why the RMSD fluctuations are very similar for all of the peptides to the RMSD of Be-HLA-DP2, simply because the type of peptide does not matter.

If the same procedure is applied to the structures built under the scenario “peptide→(Be+protein)”, the effect is opposite (Figure 6.D). The binding of all peptides onto Be-HLA-DP2 complex reduces the RMSD associated with helix 50–86.B. This response is just opposite to the increase of RMSD of helix 50–86.B upon binding of “natural” peptides in absence of the Be ion. The most striking observation is that, in presence of the Be ion, peptides from the “strong”, “weak”, and “DR” sets induce practically the same conformational changes in helix 50–86.B: reduce both the level of saturation and fluctuations compared to HLA-DP2 bound to the “natural” peptides. The same is valid for “natural” peptides to lesser extent. It can be viewed as the binding is rigidifying the pocket. Since this change is completely opposite to the change induced by the binding of ‘natural” peptides in the absence of Be, it can be speculated that it might trigger a different signaling cascade.

#### 2. Analyzing the changes in the width of peptide-binding pocket

To analyze the change in the size of peptide-binding pocket upon the binding of the Be ion and peptides in the four different sets, we monitored the change in the distances between five selected positions within the helices that form the “walls” of the pocket (see Figure 4). The results are shown in Figure 6 where the position between CA atoms for each of the 5 pairs of residues (see Figure 4, Table 1) averaged for the last 0.4 ns of MD simulations within the corresponding set are plotted. To demonstrate the distance fluctuations, we also included two standard deviations of the average data as error bars on the graphs.

**Figure 7:**
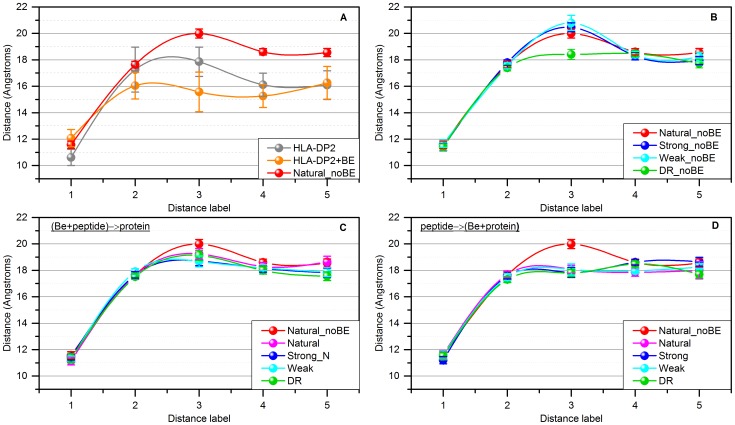
Average distances between the CA atoms of residues forming peptide-binding pocket. Error bar illustrated as ± two values of standard deviation.

As in the previous paragraph, we begin the modeling with simulations of peptide-free HLA-DP2, along with HLA-DP2 loaded with a “natural” peptide and with a Be ion. The results are shown in Figure 7.A. It can be seen that in the case of free HLA-DP2, the distance between the helices forming the gate of the binding pocket fluctuates at each positions by about 4 Å. Adding the Be ion narrows the gate at positions D3–D5, while the fluctuations remain almost the same as in the free HLA-DP2. However, when the “natural” peptides are simulated with HLA-DP2, the trend is the opposite: opening the pocket at positions D3–D5 while reducing the fluctuations. Combined with the observations made in the previous section, these results indicate that the binding of natural peptides to HLA-DP2 makes the binding pocket wider while rigidifying it.

Figure 7.B shows the results for the four sets of peptides in the absence of the Be ion. The calculated distances between the helices forming the gate of the binding pocket are almost identical for “natural”, “strong”, and “weak” peptide sets. At first, this may be considered as a contradiction of the observation made about the RMSD of helix 50–86.B in the previous section. However, the analysis of the other helix forming the gate indicates that the “strong” and “weak” peptides in the absence of Be only deform the pocket without changing its width. This indicates that the presence of the Be ion may be an important factor for regulating the conformational changes of the gate of the peptide binding pocket.

The simulations of the complexes with HLA-DP2 loaded with the four sets of peptides with the Be ion using structures delivered via the “(Be+peptide)→protein” scenario are shown in Figure 7.C. For comparison, the figure also shows the results for the HLA-DP2 with the “natural” peptides without Be. The data shows that there is no difference in the size of the pocket between each of the sets of peptides with the Be ion and the “natural” peptide without the ion. The same similarities were shown previously while analyzing the binding affinity (Figure 5.B) and the conformational changes of helix 50–86.B (Figure 6.C).

The same calculations for the “peptide→(Be+protein)” scenario (Fig 7.D) show that all sets of peptides bound to the HLA-DP2 cause the same conformational changes. This change is distinctly smaller at the distance D3 as compared to HLA-DP2 loaded with “natural” peptides. In contrast, the length at distance markers D4 and D5 is slightly longer in comparison with the HLA-DP2 protein in complex with the “natural” peptides. This is a distinctive signature of the binding effects caused by binding of each of the peptides onto HLA-DP2 already loaded with the Be ion. These conformational changes are significantly different from the conformational changes induced by the binding of “natural” peptides in the absence of the Be ion and perhaps will trigger a different signaling cascade.

### pKa calculation of ionizable residues due to small peptides and/or Be ion binding to HLA-DP2

**Figure 8:**
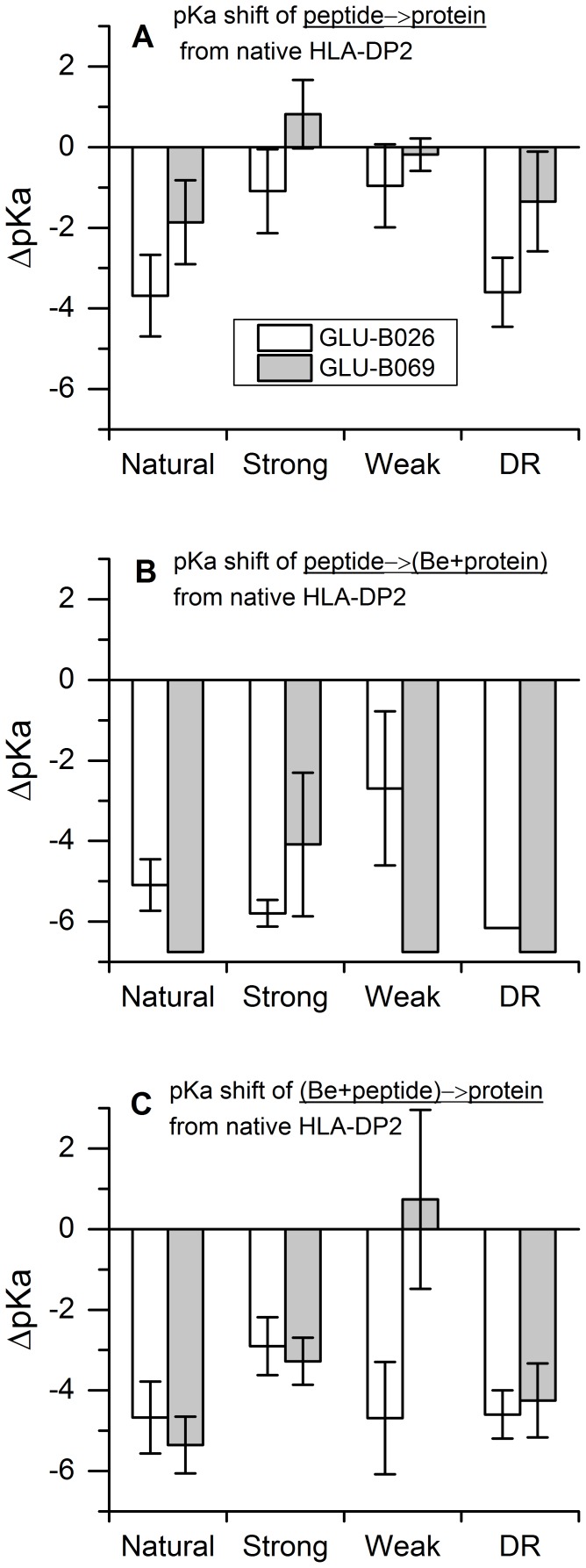
The pKa shift of two ionizable residues βGlu26 and βGlu69 on (A) HLA-DP2 binds to peptides (Four types as “natural”, “strong”, “weak” and “DR”, the same to the followings) (B) peptides bind to the complex (Be+protein) (C) (Be+peptides) complex binds to HLA-DP2 protein from pKa of native unbond protein. The pKa value was calculated as the average of 10 structures for each type of peptides.

Binding frequently causes ionization changes, typically referred to as proton uptake/release [44], [45] and thus affects the electrostatic component of the binding free energy [46]. To investigate the effects of small peptides and the Be ion binding to HLA-DP2 on the ionization states of titratable groups, we carried out a pKa calculation using the MCCE program [41], [42], [43]. The pKa's of all ionizable residues of HLA-DP2 were calculated, and the protonation changes caused by the peptide binding, especially for the residues within the pocket, were analyzed. As described in the methods section, 120 structures were subjected to the pKa calculations. 40 of them were the HLA-DP2 bound with a peptide in the absence of the Be ion and 40 of them were the protein-Be complex bound to a small peptide and the rest were the protein bound to the Be-peptide complex. They were also divided into 4 groups by the peptide categories: “DR”, “natural”, “strong”, and “weak”. Also, for each group, we obtained the average pKa values of 10 structures along with the standard error and the calculated pKa shift of them from the native HLA-DP2 protein (PDB ID: 3LZM) (see Figure 8).

We found two acidic residues, βGlu26 and βGlu69, always experience a large pKa change that is caused by the peptide or Be ion binding, although several other ionizable residues also showed slight pKa shifts. Figure 8 shows the pKa shifts for βGlu26 (blue bar) and βGlu69 (red bar) as HLA-DP2 binds four different types of peptides in presence/absence of Be ion. The pKa values for βGlu26 and βGlu69 on free HLA-DP2 are 6.2 and 6.8 respectively, i.e. they are almost neutral at pH = 7. We found that in the absence of the Be ion, the pKa shift that was caused by the binding of most of the types of peptides is less than 2.0 for βGlu26 and βGlu69 although the pKa shift for βGlu26 is about 3.5 for the “natural” and “DR” peptides. However, when the Be ion participates in the binding, both pKa shifts increase significantly and this results in deprotonation (both glutamic acids become fully charged). Especially when the Be ion binds with HLA-DP2 first and then the complex binds to the peptide, most of the pKa's were lowered more than 5.0, which indicates that the glutamic acids are fully deprotonated due to the Be ion. This is another observation in favor of the hypothesis that the Be ion is crucial for the HLA-DP2 response to the binding and the nature of the binding peptide is a secondary factor. However, since the binding affinity strongly differs among peptides, it is expected that “strong” peptides will dominate.

## Conclusions

Three questions were addressed in this study: (a) Where does the Be ion bind?; (b) What is the sequence of the events for forming the Be-HLA-DP2-peptide complex?; and (c) What is the specific “signature” of the effects induced by CBD-causing peptides and Be?

The putative Be position was predicted by the BION server [30] and it was found to be in the previously suggested acidic pocket composed of three acidic residues, Glu26, Glu68 and Glu69 [20], [23], [26]. The predicted Be position was tested in MD stimulations both with and without peptides and it was observed that the Be ion stays within the pocket and does not drift away within 1 ns of simulations. As the manuscript was under review, the Be position was experimentally determined [1] and our predicted Be position was in close proximity to the experimental one (∼3.2 Å).

Our calculations show that for the scenario “(Be+peptide)→protein” the conformational changes of the HLA-DP2 protein induced by the peptides with the attached Be ion are identical to those caused by “natural” peptides alone. This indicates that if this scenario occurs in CBD development, then the common TC receptors should recognize the complex and exert the “normal” immune response. However, the possibility of this scenario is negligibly small because of a) the decreased binding affinity between (Be+pepide) and protein and b) small peptides are very flexible in the free state (unbound state) and holding the Be ion is quite unlikely. In addition, since the binding energies for all four sets of peptides in the scenario “(Be+peptide)→protein were much worse when compared with the “peptide→(Be+protein)” scenario, it was concluded that the “peptide→(Be+protein)” scenario is more likely to occur in CBD.

Comparing the calculated effects across the four sets of peptides, it can be suggested that the specific “signature” of CBD is complex. The key player is the Be ion, which, when binds to the acidic pocket of HLA-DP2, changes the conformational properties of the peptide binding pocket. The peptide affinity is unaffected by the Be presence, but once the peptides bind to the pocket pre-loaded with the Be ion, they all induce similar conformational and ionization changes. These changes are distinctively different from the changes caused by the binding of “natural” peptides to the HLA-DP2 in absence of Be and, thus, are expected to trigger a completely different cascade of immune reactions which then leads to CBD. This conclusion is supported by the experimental results suggesting that in patients with CBD, the Be-specific CD4+T cells (Vβ5+CD4+T cell [47]) are responsible for the complex recognition and immune response propagation [48], [49], [50]. Furthermore, recent experimental work suggests that Be-specific T cells recognize the peptide-Be-HLA-DP2 complex via an unconventional docking topology [51], which in our work is referred to as a different conformational change of HLA-DP2 in CBD as compared with the normal immune response. Thus, we hypothesize that the peptide binding to the Be doped HLA-DP2 causes CBD development as recently confirmed experimentally [1].

## Supporting Information

Material S1 **Sequence alignment of the “natural”, “strong”, and “weak” peptides.**
(RTF)

## References

[1] Clayton GM, Wang Y, Crawford F, Novikov A, Wimberly BT, et al (2014) Structural Basis of Chronic Beryllium Disease: Linking Allergic Hypersensitivity and Autoimmunity. Cell 158: 132–142. 24995984 10.1016/j.cell.2014.04.048 PMC4269484

[2] Van Dyke MV, Martyny JW, Mroz MM, Silveira LJ, Strand M, et al (2011) Exposure and genetics increase risk of beryllium sensitisation and chronic beryllium disease in the nuclear weapons industry. Occupational and environmental medicine 68: 842–848. 21460389 10.1136/oem.2010.064220 PMC4347849

[3] Silveira LJ, McCanlies EC, Fingerlin TE, Van Dyke MV, Mroz MM, et al (2012) Chronic beryllium disease, HLA-DPB1, and the DP peptide binding groove. The Journal of Immunology 189: 4014–4023. 22972925 10.4049/jimmunol.1200798 PMC4347851

[4] Bowerman NA, Falta MT, Mack DG, Kappler JW, Fontenot AP (2011) Mutagenesis of beryllium-specific TCRs suggests an unusual binding topology for antigen recognition. The Journal of Immunology 187: 3694–3703. 21873524 10.4049/jimmunol.1101872 PMC3178675

[5] Díaz G, Cañas B, Vazquez J, Nombela C, Arroyo J (2005) Characterization of natural peptide ligands from HLA-DP2: new insights into HLA-DP peptide-binding motifs. Immunogenetics 56: 754–759. 15565337 10.1007/s00251-004-0735-5

[6] Wang Y, Dai S (2013) Structural basis of metal hypersensitivity. Immunologic research 55: 83–90. 22983897 10.1007/s12026-012-8351-1 PMC4040395

[7] Welch LS, Ringen K, Dement J, Bingham E, Quinn P, et al (2013) Beryllium disease among construction trade workers at department of Energy nuclear sites. American journal of industrial medicine 56: 1125–1136. 23794247 10.1002/ajim.22202

[8] Van Dyke MV, Martyny JW, Mroz MM, Silveira LJ, Strand M, et al (2011) Risk of chronic beryllium disease by HLA-DPB1 E69 genotype and beryllium exposure in nuclear workers. American journal of respiratory and critical care medicine 183: 1680–1688. 21471109 10.1164/rccm.201002-0254OC PMC3136994

[9] Dai S, Falta MT, Bowerman NA, McKee AS, Fontenot AP (2013) T cell recognition of beryllium. Current opinion in immunology 25: 775–780. 23978481 10.1016/j.coi.2013.07.012 PMC3883670

[10] Thomas CA, Deubner DC, Stanton ML, Kreiss K, Schuler CR (2013) Long-Term Efficacy of a Program to Prevent Beryllium Disease. American journal of industrial medicine 56: 733–741. 23450749 10.1002/ajim.22175

[11] Mayer A, Brazile W, Erb S, Barker E, Miller C, et al (2013) Developing Effective Health and Safety Training Materials for Workers in Beryllium-Using Industries. Journal of Occupational and Environmental Medicine 55: 746–751. 23787563 10.1097/JOM.0b013e3182972f1b

[12] Salvator H, Gille T, Hervé A, Bron C, Lamberto C, et al (2013) Chronic beryllium disease: azathioprine as a possible alternative to corticosteroid treatment. European Respiratory Journal 41: 234–236. 23277520 10.1183/09031936.00095712

[13] Maier LA, Barkes BQ, Mroz M, Rossman MD, Barnard J, et al (2012) Infliximab therapy modulates an antigen-specific immune response in chronic beryllium disease. Respiratory medicine 106: 1810–1813. 22974830 10.1016/j.rmed.2012.08.014 PMC4347848

[14] Seidler A, Euler U, Müller-Quernheim J, Gaede K, Latza U, et al (2012) Systematic review: progression of beryllium sensitization to chronic beryllium disease. Occupational medicine 62: 506–513. 22705916 10.1093/occmed/kqs069

[15] Chain JL, Martin AK, Mack DG, Maier LA, Palmer BE, et al (2013) Impaired function of ctla-4 in the lungs of patients with chronic beryllium disease contributes to persistent inflammation. The Journal of Immunology 191: 1648–1656. 23851684 10.4049/jimmunol.1300282 PMC3750981

[16] Bowerman NA, Falta MT, Mack DG, Wehrmann F, Crawford F, et al.. (2014) Identification of Multiple Public TCR Repertoires in Chronic Beryllium Disease. The Journal of Immunology: 1400007. 10.4049/jimmunol.1400007 PMC4011988 24719461

[17] Yucesoy B, Johnson VJ, Lummus ZL, Kashon ML, Rao M, et al (2014) Genetic Variants in the Major Histocompatibility Complex Class I and Class II Genes Are Associated With Diisocyanate-Induced Asthma. Journal of Occupational and Environmental Medicine 56: 382–387. 24709764 10.1097/JOM.0000000000000138 PMC4572490

[18] Morris D, Fernando M, Taylor K, Chung S, Nititham J, et al (2014) MHC associations with clinical and autoantibody manifestations in European SLE. Genes and immunity 10.1038/gene.2014.6 PMC4102853 24598797

[19] Pimentel-Santos F, Matos M, Ligeiro D, Mourão A, Ribeiro C, et al (2013) HLA alleles and HLA-B27 haplotypes associated with susceptibility and severity of ankylosing spondylitis in a Portuguese population. Tissue Antigens 82: 374–379. 24498993 10.1111/tan.12238

[20] Richeldi L, Sorrentino R, Saltini C (1993) HLA-DPB1 glutamate 69: a genetic marker of beryllium disease. Science 262: 242–244. 8105536 10.1126/science.8105536

[21] Fontenot AP, Torres M, Marshall WH, Newman LS, Kotzin BL (2000) Beryllium presentation to CD4+ T cells underlies disease-susceptibility HLA-DP alleles in chronic beryllium disease. Proc Natl Acad Sci U S A 97: 12717–12722. 11050177 10.1073/pnas.220430797 PMC18830

[22] Bill JR, Mack DG, Falta MT, Maier LA, Sullivan AK, et al (2005) Beryllium presentation to CD4+ T cells is dependent on a single amino acid residue of the MHC class II beta-chain. J Immunol 175: 7029–7037. 16272364 10.4049/jimmunol.175.10.7029

[23] Dai S, Murphy GA, Crawford F, Mack DG, Falta MT, et al (2010) Crystal structure of HLA-DP2 and implications for chronic beryllium disease. Proc Natl Acad Sci U S A 107: 7425–7430. 20356827 10.1073/pnas.1001772107 PMC2867715

[24] Patronov A, Dimitrov I, Flower DR, Doytchinova I (2011) Peptide binding prediction for the human class II MHC allele HLA-DP2: a molecular docking approach. BMC structural biology 11: 32. 21752305 10.1186/1472-6807-11-32 PMC3146810

[25] Doytchinova I, Petkov P, Dimitrov I, Atanasova M, Flower DR (2011) HLA-DP2 binding prediction by molecular dynamics simulations. Protein Science 20: 1918–1928. 21898654 10.1002/pro.732 PMC3267955

[26] Falta MT, Pinilla C, Mack DG, Tinega AN, Crawford F, et al (2013) Identification of beryllium-dependent peptides recognized by CD4+ T cells in chronic beryllium disease. The Journal of experimental medicine 210: 1403–1418. 23797096 10.1084/jem.20122426 PMC3698527

[27] Amicosante M, Sanarico N, Berretta F, Arroyo J, Lombardi G, et al (2001) Beryllium binding to HLA-DP molecule carrying the marker of susceptibility to berylliosis glutamate β69. Human immunology 62: 686–693. 11423174 10.1016/s0198-8859(01)00261-0

[28] Scott BL, Wang Z, Marrone BL, Sauer NN (2003) Potential binding modes of beryllium with the class II major histocompatibility complex HLA-DP: a combined theoretical and structural database study. Journal of inorganic biochemistry 94: 5–13. 12620668 10.1016/s0162-0134(02)00628-1

[29] Humphrey W, Dalke A, Schulten K (1996) VMD: visual molecular dynamics. J Mol Graph 14: 27–38 – 10.1016/0263-7855(96)00018-5 8744570

[30] Petukh M, Kimmet T, Alexov E (2013) BION web server: predicting non-specifically bound surface ions. Bioinformatics 29: 805–806. 23380591 10.1093/bioinformatics/btt032 PMC3597141

[31] Li C, Petukh M, Li L, Alexov E (2013) Continuous development of schemes for parallel computing of the electrostatics in biological systems: Implementation in DelPhi. Journal of computational chemistry 10.1002/jcc.23340 PMC3707979 23733490

[32] Petukh M, Zhenirovskyy M, Li C, Li L, Wang L, et al (2012) Predicting Nonspecific Ion Binding Using DelPhi. Biophys J 102: 2885–2893. 22735539 10.1016/j.bpj.2012.05.013 PMC3379622

[33] Pettersen EF, Goddard TD, Huang CC, Couch GS, Greenblatt DM, et al (2004) UCSF Chimera–a visualization system for exploratory research and analysis. J Comput Chem 25: 1605–1612. 15264254 10.1002/jcc.20084

[34] Phillips JC, Braun R, Wang W, Gumbart J, Tajkhorshid E, et al (2005) Scalable molecular dynamics with NAMD. Journal of computational chemistry 26: 1781–1802. 16222654 10.1002/jcc.20289 PMC2486339

[35] Teng S, Madej T, Panchenko A, Alexov E (2009) Modeling effects of human single nucleotide polymorphisms on protein-protein interactions. Biophysical journal 96: 2178–2188. 19289044 10.1016/j.bpj.2008.12.3904 PMC2717281

[36] Zhang Z, Teng S, Wang L, Schwartz CE, Alexov E (2010) Computational analysis of missense mutations causing Snyder-Robinson syndrome. Human mutation 31: 1043–1049. 20556796 10.1002/humu.21310 PMC2932761

[37] Zhang Z, Miteva MA, Wang L, Alexov E (2012) Analyzing effects of naturally occurring missense mutations. Computational and mathematical methods in medicine 2012 10.1155/2012/805827 PMC3346971 22577471

[38] Nishi H, Tyagi M, Teng S, Shoemaker BA, Hashimoto K, et al (2013) Cancer missense mutations alter binding properties of proteins and their interaction networks. PLoS One 8: e66273. 23799087 10.1371/journal.pone.0066273 PMC3682950

[39] Li M, Petukh M, Alexov E, Panchenko AR (2014) Predicting the Impact of Missense Mutations on Protein-Protein Binding Affinity. Journal of Chemical Theory and Computation 10.1021/ct401022c PMC3985714 24803870

[40] Zhang Z, Wang L, Gao Y, Zhang J, Zhenirovskyy M, et al (2012) Predicting folding free energy changes upon single point mutations. Bioinformatics 28: 664–671. 22238268 10.1093/bioinformatics/bts005 PMC3289912

[41] Alexov E, Gunner M (1997) Incorporating protein conformational flexibility into the calculation of pH-dependent protein properties. Biophysical journal 72: 2075–2093. 9129810 10.1016/S0006-3495(97)78851-9 PMC1184402

[42] Alexov E, Gunner M (1999) Calculated protein and proton motions coupled to electron transfer: electron transfer from QA-to QB in bacterial photosynthetic reaction centers. Biochemistry 38: 8253–8270. 10387071 10.1021/bi982700a

[43] Georgescu RE, Alexov EG, Gunner MR (2002) Combining conformational flexibility and continuum electrostatics for calculating pK(a)s in proteins. Biophys J 83: 1731–1748. 12324397 10.1016/S0006-3495(02)73940-4 PMC1302268

[44] Onufriev AV, Alexov E (2013) Protonation and pK changes in protein–ligand binding. Quarterly reviews of biophysics 46: 181–209. 23889892 10.1017/S0033583513000024 PMC4437766

[45] Petukh M, Stefl S, Alexov E (2013) The Role of Protonation States in Ligand-Receptor Recognition and Binding. Current pharmaceutical design 19: 4182. 23170880 10.2174/1381612811319230004 PMC3625499

[46] Zhang Z, Witham S, Alexov E (2011) On the role of electrostatics in protein-protein interactions. Phys Biol 8: 035001. 21572182 10.1088/1478-3975/8/3/035001 PMC3137121

[47] Bowerman N, Falta M, Mack D, Crawford F, Kappler J, et al (2013) Characterizing the T cell receptor repertoire of beryllium-responsive CD4 T cells (P5021). The Journal of Immunology 190: 110.111.

[48] Fontenot AP, Falta MT, Freed BM, Newman LS, Kotzin BL (1999) Identification of pathogenic T cells in patients with beryllium-induced lung disease. J Immunol 163: 1019–1026. 10395700

[49] Fontenot AP, Keizer TS, McCleskey M, Mack DG, Meza-Romero R, et al (2006) Recombinant HLA-DP2 binds beryllium and tolerizes beryllium-specific pathogenic CD4+ T cells. The Journal of Immunology 177: 3874–3883. 16951350 10.4049/jimmunol.177.6.3874

[50] Martin AK, Mack DG, Falta MT, Mroz MM, Newman LS, et al (2011) Beryllium-specific CD4<sup>+</sup> T cells in blood as a biomarker of disease progression. Journal of allergy and clinical immunology 128 1100-1106: e1105. 10.1016/j.jaci.2011.08.022 PMC3205205 21943943

[51] Bowerman N, Falta M, Mack D, Kappler J, Fontenot A (2011) Beryllium-specific T cells adopt an unusual binding topology for antigen recognition. The Journal of Immunology 186: 100.125. 10.4049/jimmunol.1101872 PMC3178675 21873524

