# Supplementary material for: Chronic Beryllium Disease: Revealing the Role of Beryllium Ion and Small Peptides Binding to HLA-DP2
Source: PLoS One. 2014 Nov 4;9(11):e111604. doi: 10.1371/journal.pone.0111604 (PMC4219729; doi:10.1371/journal.pone.0111604)
Supplement: Material S1 — Sequence alignment of the “natural”, “strong”, and “weak” peptides. (RTF) [file pone.0111604.s001.rtf]

Materials S1

“Natural” peptide alignments 

PDB
3LQZ	Sequence	1	2	3	4	5	6	7	8	9	10	
Natural Peptide Template 	 RK FHYLPFLPST GGS	
F	
H	
Y	
L	
P	
F	
L	
P	
S	
T	


Natural 
Peptide
 Number	Sequence	1	2	3	4	5	6	7	8	9	10	
1	DEKK
FWGKYLYEIV
AR	F	W	G	K	Y	L	Y	E	I	V	
2	LGE YGFQNALIV
R	Y	G	F	Q	N	A	L	I	V	R	
3	SIVHPS YNSNTLNND IMLIK	Y	N	S	N	T	L	N	N	D	I	
4	GGEP LSYTRFSLA RQVDG	L	S	Y	T	R	F	S	L	A	R	
5	YA YDGKDYIALK
	Y	D	G	K	D	Y	I	A	L	K	
6	FRK FHYLPFLPS
TE	F	H	Y	L	P	F	L	P	S	T	
7	YA 
FLGAKFKTS
AQ	F	L	G	A	K	F	K	T	S	A	
8	SVH IYDKAFITV KHRK	I	Y	D	K	A	F	I	T	V	K	
9	RVPPEEH PVLLTEAPI NPK	P	V	L	L	T	E	A	P	I	N	
10	KNV VKIQKHVTF NQVKG	V	K	I	Q	K	H	V	T	F	N	


“Strong” peptide alignments

Strong
Peptide Number	PDB ID	1	2	3	4	5	6	7	8	9	10	
												
1	HU1	M	W	A	D	L	L	E	L	I	D	
2	HU31	F	V	D	D	L	F	E	T	I	F	
3	HU35	F	V	D	D	L	F	E	T	L	F	
4	HU33	F	V	D	D	L	F	E	T	L	F	
5	HU2	C	F	L	D	L	L	E	L	L	I	
6	HU25	F	V	D	D	L	F	E	T	L	L	
7	HU3	A	W	A	D	L	L	E	L	I	D	
8	HU39	F	W	L	P	L	L	E	K	V	Y	
9	HU9	F	W	L	R	L	L	E	L	T	W	
10	HU21	Y	C	I	D	L	L	E	R	L	A	


“Weak” peptide alignments 

Weak
Peptide Number	
PDB ID	
1	
2	
3	
4	
5	
6	
7	
8	
9	
10	
												
1	HU8	C	W	L	S	L	L	E	Y	L	L	
2	HU20	F	W	A	R	L	L	E	R	L	F	
3	HU27	L	C	A	D	L	F	E	R	V	P	
4	HU18	H	W	L	D	L	F	R	L	L	G	
5	HU23	F	W	T	P	L	L	E	S	L	A	
6	HU19	C	W	V	D	L	I	I	S	S	S	
7	HU12	C	W	L	D	L	L	L	A	A	L	
8	HU13	L	W	I	L	L	L	E	T	S	L	
9	HU29	W	W	Q	D	L	F	R	I	V	L	
10	HU30	L	K	V	D	L	L	E	Q	T	K	


“DR” peptide alignments

PDB
3LQZ	Sequence	1	2	3	4	5	6	7	8	9	10	
Natural Peptide Template 	RK FHYLPFLPST GGS	
F	
H	
Y	
L	
P	
F	
L	
P	
S	
T	


PDB
1KG0	Sequence	1	2	3	4	5	6	7	8	9	10	
DR Peptide Template	PK YVKQNTLKLAT	
Y	
V	
K	
Q	
N	
T	
L	
K	
L	
A	


DR
Peptide Number	Sequence	1	2	3	4	5	6	7	8	9	10	

1	SMRYQSLIPR  LVEFF	
S	
M	
R	
Y	
Q	
S	
L	
I	
P	
R	

2	QWS YMHISGQDAS EY	
Y	
M	
H	
I	
S	
G	
Q	
D	
A	
S	

3	VDCYINLGAR  WSLDY	
V	
D	
C	
Y	
I	
N	
L	
G	
A	
R	

4	TSL  YNLRRGTALA	
Y	
N	
L	
R	
R	
G	
T	
A	
L	
A	

5	FRKQNPDIVI YQYMD DLYVG	
F	
R	
K	
Q	
N	
P	
D	
I	
V	
I	

6	KA  LENKKKQLGA  GGKN	
L	
E	
N	
K	
K	
K	
Q	
L	
G	
A	

7	
RV VINKDTTIII	
V	
I	
N	
K	
D	
T	
T	
I	
I	
I	

8	T  IRLTAADHRQ  LQLS	
I	
R	
L	
T	
A	
A	
D	
H	
R	
Q	

9	 PYY  TGEHAKAIGN	
T	
G	
E	
H	
A	
K	
A	
I	
G	
N	

10	MKV VIVTSVASLL DASIQFQK	
V	
I	
V	
T	
S	
V	
A	
S	
L	
L	
